# Continuity and locum use for acute consultations: observational study of subsequent workload

**DOI:** 10.3399/BJGP.2024.0312

**Published:** 2025-01-28

**Authors:** Harshita Kajaria-Montag, Stefan Scholtes, Denis Pereira Gray, Kate Sidaway-Lee, Michael Freeman, Philip Evans

**Affiliations:** Kelley School of Business, Indiana University, Bloomington, IN, US.; Judge Business School, University of Cambridge, Cambridge, UK.; St Leonard’s Research Practice, Exeter; emeritus professor, University of Exeter Medical School, University of Exeter, Exeter, UK.; St Leonard’s Research Practice, Exeter, UK.; Technology and Operations Management, INSEAD Asia Campus, Singapore.; University of Exeter Medical School, University of Exeter, Exeter; consultant, St Leonard’s Research Practice, Exeter, UK.

**Keywords:** primary health care, workload, hospitals, continuity of care, general practice

## Abstract

**Background:**

Workload is probably the biggest challenge facing general practice and little is known about any modifiable factors. For GPs, both continuity and locum status are associated with differences in outcomes.

**Aim:**

To determine whether practice and hospital workload after an index acute consultation depend on the type of GP consulted (locums and practice GPs with [regular] and without [non-regular] continuity, and locums).

**Design and setting:**

An observational, cross-sectional analysis of consultation-level data from English general practices from the Clinical Practice Research Datalink from 2015 to 2017.

**Method:**

Antibiotic prescription was used as a marker for acute consultations with regression models to calculate adjusted relative risks for emergency department consultations and admissions, outpatient referrals, and test ordering, as well as the patients’ GP reconsultation interval following consultations with the three types of GP.

**Results:**

After adjustment, consultations with antibiotic prescriptions with regular GPs with continuity were associated with fewer subsequent hospital admissions and lower emergency department use but higher outpatient referrals relative to locums and non-regular GPs. Locums ordered tests less often (relative risk [RR] −24.3%, 95% confidence interval [CI] = −27.3 to −21.2) than regular GPs whereas non-regular GPs ordered tests more often (RR 19.1%, 95% CI = = 16.4 to 21.8). Patients seeing their regular GP had on average a 9% longer (95% CI = 8 to 10) reconsultation interval than if they saw any other GP.

**Conclusion:**

The differences in outcomes were associated more with having continuity than with GP locum status. Seeing a GP with whom the patient had continuity of care was associated with reduced workload within the practice and in hospital.

## Introduction

Increasing primary and secondary care workloads are a serious challenge within the NHS and health systems internationally. Explanations include an ageing population, pressures caused by the COVID-19 pandemic, and increasing multimorbidity. However, there may also be factors within general practices contributing to increases in workload.

GP partners and permanently employed salaried GPs may have continuity of care with patients. Continuity has been shown to be associated with better health outcomes for patients including greater patient satisfaction,^1^ improved adherence to medical advice,^2^ better preventive care,^3^ better prescribing,^4^ lower overall costs for health systems,^5^ and even longer life expectancy.^6^^,^^7^ Continuity has also been shown to be associated with lower hospital admissions,^8^^,^^9^ fewer accident and emergency department (A&E) visits,^5^ and a longer reconsultation interval.^10^ It has been estimated that GPs in the top decile for continuity saved their practices up to 5.2% of consultations among more frequent attenders.^10^ However, continuity of care has been falling nationally since at least 2012.^11^

Locum GPs are fully qualified GPs who are self-employed or on short-term contracts with practices. They cover periods of leave of permanent GPs or staffing gaps.^12^ Locums comprised 3.2% of the GP workforce by full-time equivalents (1046 of 33 133 total GP full-time equivalents) in December 2017,^13^ and were found to have provided 11% of appointments for some specific acute conditions in 2013–2015.^14^ The use of locums in English general practices remained fairly stable from 2017 to 2020.^13^

Grigoroglou *et al* found that after consultations with locums, patients were more likely to visit A&E departments but less likely to reconsult within a week compared with permanent GPs.^15^ However, this study did not examine whether consulting a practice GP with continuity differed from a consultation with a locum. There may also have been differences in case mix between patients who saw a locum compared with a permanent GP.

To distinguish whether there are differences in outcomes with locums when there is pre-existing continuity, in the current study GPs were divided into three groups:

- locums;
- GP partners and salaried GPs with continuity (regular GPs); and
- GP partners and salaried GPs without continuity (non-regular GPs).

**Table:** How this fits in

Continuity of GP care has been shown to be associated with a wide range of benefits for patients and the health system. Evidence is emerging that there are also benefits for practices, as overall practice workload was reduced by up to 5.2% when patients had GP continuity. Locums provide around 3% of consultations and this study found that for some key outcomes they did not differ from practice GPs with whom patients have no continuity but did differ from practice GPs with continuity. GP continuity, with the exception of the use of investigations and outpatient referrals, was associated with reduced overall practice and hospital workload for the consultations studied, all of which involved prescribing of an antibiotic.

Regular GPs were those whom individual patients had seen most often in the past 2 years. The study used consultations with antibiotic prescriptions to reduce differences in case mix and examined several subsequent outcomes related to GP and hospital workload.

## Method

### Data

A cross-sectional analysis was performed of face-to-face consultations between patients and GPs and associations evaluated between the type of consulting GP — regular GP, non-regular GP, or locum — and subsequent primary and secondary care use following an index consultation for an acute condition. Data were used from the Clinical Practice Research Datalink (CPRD), a large database of anonymised consultation-level general practice electronic health records that is representative of the UK population in terms of age, sex, and ethnicity.^16^ Exclusion criteria were applied as in Kajaria-Montag *et al*^10^ to ensure a homogeneous, clean, and reliable sample.

The full sample included face-to-face consultations between fully registered GPs and adult patients (aged ≥18 years) who were registered with their practice between 1 January 2015 and 31 December 2017.

CPRD lacks appointment scheduling data, making it difficult to distinguish between acute and routine appointments. As acuity is likely to be different between GP types, for index consultations the study used a subset of all urgent appointments by using the prescription of any antibiotic during the consultation as a marker for acuity.

### Measures

The main independent variable was a categorical variable with three categories that identified whether the index consultation was with a locum GP, a regular GP, or a non-regular GP. Locums were identified through a staff identifier role within CPRD. Regular or non-regular GPs could be partners or salaried doctors but not locums or GP registrars. The patient’s regular GP was the GP with whom the patient had the greatest number of face-to-face consultations during the 2 years preceding the index consultation. To ensure reliability in calculations and to remain consistent with the literature, only face-to-face GP consultations were considered and ≥3 consultations within the 2 years preceding the index consultation was required to demonstrate continuity. To estimate the effect of care continuity only when appointments could have been with a patient’s regular doctor, appointments were excluded as index consultations if the regular GP did not see any patients in that particular week. The study also excluded the first 2 years of the patient’s registration with the practice to ensure that the patient had sufficient time to identify a regular GP.

The relationship was studied between the type of consulting GP and healthcare utilisation metrics following the index consultation. Specifically, several secondary care utilisation measures were considered by linking CPRD data to Hospital Episode Statistics and general practice measures calculated based on the data in CPRD. Binary variables included same-day A&E visit, 7-day A&E visit (excluding same day), same-day emergency hospital admission, 7-day emergency hospital admission (excluding same day), 7-day A&E visit without admission, outpatient referrals, and investigations ordered (index-consultation linked). Reconsultation interval was a continuous variable and was calculated as the number of days until the patient’s next face-to-face GP consultation with any GP.

### Control variables

The relationship between the type of doctor a patient saw and the dependent variables were likely to be confounded by several factors such as demographics, a patient’s consultation history, temporal factors, and practice-level factors. Within the analysis, deprivation (using Index of Multiple Deprivation 2015 quintiles), sex, age, comorbidities, total practice demand, patient consultation frequency, practice factors, year, seasonality, and day of the week were controlled for (see Supplementary Table S1).

### Statistical analysis

The relationship between a binary system utilisation measure and the doctor category was estimated by a multivariate modified Poisson regression method^17^^,^^18^ using indicator variables for the locum and non-regular GP categories, respectively. This method allowed the authors to estimate relative risks, with consultations with the patient’s regular GP as the reference category. The relationship between the continuous reconsultation interval was estimated using a log-linear multivariate regression model. To account for potential dependence of the error terms across patients in a practice, standard errors were clustered in all models at the practice level. Stata (version 16.1) was used to conduct the analyses.

### Robustness

To further confirm the robustness of the results, additional analyses were performed:

- using only females or only males as two different subsamples to account for potential bias that one sex might be more likely to see a locum;
- splitting the locum GP category into high and low engagement, based on the locum’s engagement with the practice in the 12 months before the index consultation;
- splitting the non-regular GP category into high and low engagement, based on the GP’s engagement with the practice in the 12 months before the consultation; and
- using additional testing-related outcome measures such as midstream specimen of urine and blood tests.

## Results

**Figure 1.**
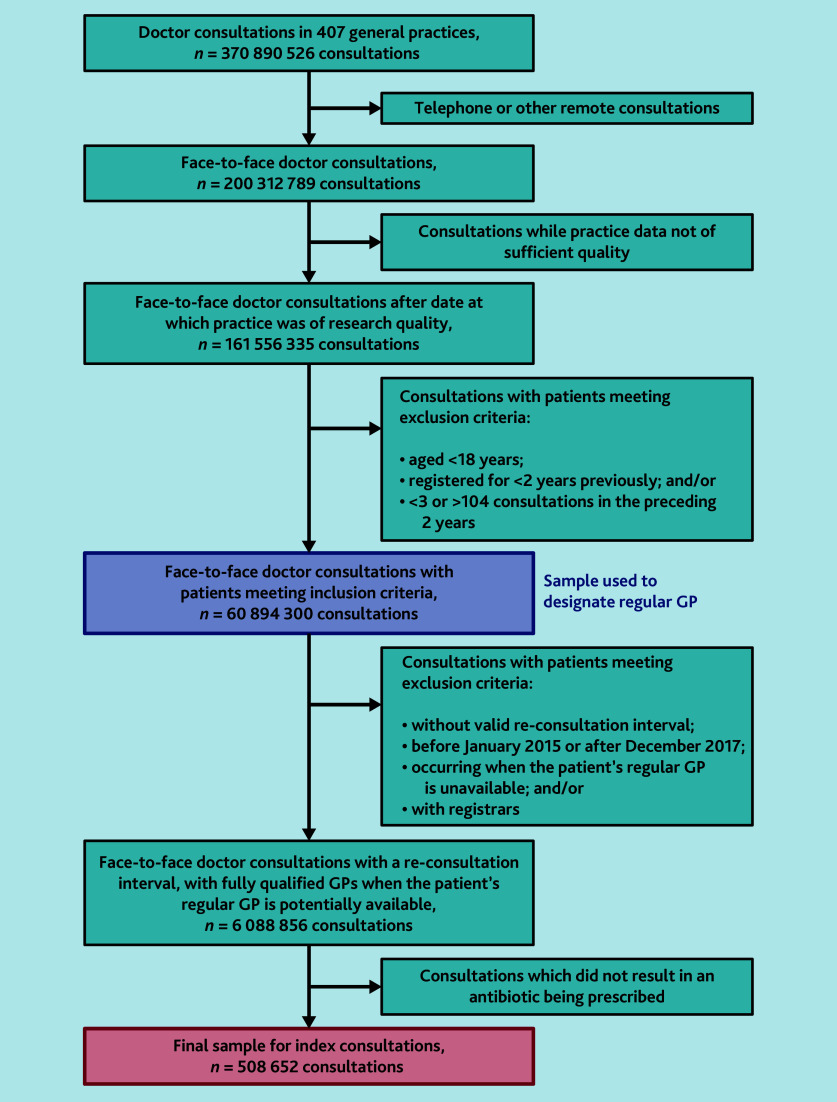
Exclusions leading to the sample for continuity calculation and then to the sample of index consultations.

After exclusions (Figure 1) the sample comprised 508 652 consultations from 222 practices, with 2854 GPs corresponding to 252 242 patients all prescribed an antibiotic during the consultation (Table 1). Of these, 198 102 (38.9%) consultations were with the patient’s regular (continuity) GP, 252 550 (49.7%) were with a non-regular practice GP, and 58 000 (11.4%) were with a locum.

**Table 1.** Characteristics of patients seen by each type of doctor^a^

**Characteristic**	**All consultations** **(*n* = 508 652)**	**Consultations with locum (*n* = 58 000)**	**Consultations with regular GP (*n* = 198 102)**	**Consultations with non-regular GP (*n* = 252 550)**
**Patients, *n***	252 242	44 851	117 856	160 489

**Age, years, *n* (%)**				
18–30	54 801 (10.8)	7622 (13.1)	16 310 (8.2)	30 869 (12.2)
31–40	53 235 (10.5)	7467 (12.9)	16 876 (8.5)	28 892 (11.4)
41–50	67 505 (13.3)	8664 (14.9)	23 969 (12.1)	34 872 (13.8)
51–60	79 769 (15.7)	9571 (16.5)	30 686 (15.5)	39 512 (15.6)
61–70	91 290 (17.9)	10 064 (17.4)	37 839 (19.1)	43 387 (17.2)
71–80	88 555 (17.4)	8527 (14.7)	39 244 (19.8)	40 784 (16.1)
81–90	58 923 (11.6)	4959 (8.6)	26 652 (13.5)	27 312 (10.8)
≥91	14 574 (2.9)	1126 (1.9)	6526 (3.3)	6922 (2.7)

**Sex, *n* (%)**				
Male	171 512 (33.7)	18 740 (32.3)	69 690 (35.2)	83 082 (32.9)

**Comorbidities, *n* (%)**				
0	93 155 (18.3)	12 633 (21.8)	29 295 (14.8)	51 227 (20.3)
1	95 210 (18.7)	11 995 (20.7)	33 284 (16.8)	49 931 (19.8)
2	84 211 (16.6)	9964 (17.2)	32 358 (16.3)	41 889 (16.6)
3	70 808 (13.9)	7569 (13.1)	29 475 (14.9)	33 764 (13.4)
4	56 531 (11.1)	5809 (10.0)	24 642 (12.4)	26 080 (10.3)
≥5	108 737 (21.4)	10 030 (17.3)	49 048 (24.8)	49 659 (19.7)

**2015 IMD quintile, *n* (%)^b^**				
1 (least deprived)	119 888 (23.6)	11 859 (20.4)	44 173 (22.3)	63 856 (25.3)
2	101 461 (19.9)	10 981 (18.9)	39 741 (20.1)	50 739 (20.1)
3	101 532 (20.0)	11 906 (20.5)	39 378 (19.9)	50 248 (19.9)
4	101 087 (19.9)	12 414 (21.4)	40 932 (20.7)	47 741 (18.9)
5 (most deprived)	84 582 (16.6)	10 832 (18.7)	33 837 (17.1)	39 913 (15.8)

a *These show numbers of consultations as some patients had >1 consultation during the study period.*

b *IMD values are missing for 65 patients corresponding to 112 consultations. IMD = Index of Multiple Deprivation.*

Regular GPs were more likely to see patients who were older (aged 61 years compared with aged 55 years for locums and aged 57 years for non-regular GPs, *P*<0.01) and had more comorbidities (Table 1). Before adjustment for age, comorbidity, and other factors, patients who saw their regular GP had a shorter reconsultation interval (Table 2).

**Table 2.** Unadjusted numbers of consultations that resulted in the outcomes investigated^a^

**Characteristic**	**All consultations**	**Consultations with locum**	**Consultations with regular GP**	**Consultations with non-regular GP**
**Patients**	252 242	44 851	117 856	160 489

**Consultations**	508 652	58 000	198 102	252 550

**Outcomes**				
Same-day A&E visit	2481 (0.49)	307 (0.53)	818 (0.41)	1356 (0.54)
Seven-day A&E visit (excluding same-day visit)	7625 (1.50)	897 (1.55)	2826 (1.43)	3902 (1.55)
Seven-day A&E visit without admission	8294 (1.63)	1047 (1.81)	2866 (1.45)	4381 (1.73)
Same-day emergency admission	856 (0.17)	103 (0.18)	307 (0.15)	446 (0.18)
Seven-day emergency admission (excluding same-day admission)	10 666 (2.10)	1120 (1.93)	4181 (2.11)	5365 (2.12)
Outpatient referrals	14 165 (2.78)	1317 (2.27)	6173 (3.12)	6675 (2.64)
Test ordering	39 612 (7.79)	3368 (5.81)	13 864 (7.00)	22 380 (8.86)
GP reconsultation interval, days, mean	60.6	66.2	57.5	61.7

a *The percentages are the percentage of consultations resulting in the particular outcome. Data are* n *(%) unless otherwise indicated. A&E = accident and emergency.*

Patients seeing locums were younger compared to those seeing regular or non-regular GPs, with fewer comorbidities (Table 1) but similar levels of A&E attendance and subsequent hospital admissions in the unadjusted analysis (Table 2).

After adjustment, subsequent A&E visits were more likely after patients consulted with locums and non-regular GPs compared with consulting their regular GP (Table 3). Relative to a consultation with the patient’s regular GP, the relative risk of a same-day emergency department visit increased by 22.3% (95% confidence interval [CI] = 2.1 to 47.0) for locum consultations and by 30.0% (95% CI = 17.7 to 43.3) for consultations with non-regular GPs. The difference between locums and non-regular GPs was not statistically significant. The effects were, however, statistically significant for A&E visits in the 6 days following the index consultation. The 7-day emergency hospital admission rate (excluding same-day admissions) was elevated by an estimated 11.1% (95% CI = 6.0 to 16.4, *P*<0.001) when patients saw a non-regular GP in their practice but not if they saw a locum.

**Table 3.** Results of the adjusted model showing RR and percentage change estimates for locums or non-regular GPs compared with regular GPs

**Characteristic**	**Locum versus regular GP, RR (95% CI)^a^**	**Non-regular GP versus regular GP, RR (95% CI)^a^**	**Difference in RRs between locums and non-regular GP, *P*-value**
**Same-day A&E visit**	22.3^b^ (2.1 to 47.0)	30.0^c^ (17.7 to 43.3)	0.49
**Seven-day A&E visit (excluding same-day consultation)**	11.9^b^ (2.5 to 22.2)	18.2^c^ (12.6 to 24.1)	0.22
**Seven-day A&E visit without admission**	20.9^c^ (11.1 to 31.7)	24.1^c^(17.9 to 30.6)	0.56
**Same-day emergency admission**	29.3 (−2.5 to 71.4)	25.7^d^(5.8 to 49.4)	0.83
**Seven-day emergency admission (excluding same-day admission)**	1.7 (−5.0 to 8.8)	11.1^c^(6.0 to 16.4)	0.01
**Outpatient referrals**	−37.7^c^ (−48.3 to −24.9)	−19.7^c^(−26.3 to −12.6)	0.01
**Test ordering**	−24.3^c^ (−27.3 to −21.2)	19.1^c^(16.4 to 21.8)	<0.001
**GP reconsultation interval**	−9.1^c^ (−12.1 to −6.0)	−9.1^c^(−10.5 to −7.5)	0.97

a *All data are RR (95% CI) except for the final row where data are the respective percentage change in the reconsultation interval (95% CI), using an ordinary least squares model with other controls included. The RR (95% CI), using a modified Poisson model with other controls included, show percentage of event occurrence when the index consultation was with a locum or a non-regular GP, relative to the patient’s regular GP.*

b P*<0.05 for risk compared with regular GP.*

c P*<0.001 for risk compared with regular GP.*

d P*<0.01 for risk compared with regular GP. A&E = accident and emergency. RR = relative risk.*

For referrals, both locums and non-regular GPs were significantly less likely to refer than the patient’s regular GP. The referral rate reduced by 19.7% (95% CI = 12.6 to 26.3) for a non-regular GP and by 37.7% (95% CI = 24.9 to 48.3) when the consultation was with a locum (Table 3). Test-ordering behaviours differed between locums and non-regular GP when compared with regular GPs, with locums requesting 24.3% fewer (95% CI = 21.2 to 27.3) and non-regular GPs ordering 19.1% more (95% CI = 16.4 to 21.8).

The reconsultation interval was 9.1% shorter when the consultation was with a locum (95% CI = 6.0 to 12.1) or non-regular GP (95% CI = 7.5 to 10.5), compared with a regular GP (Table 3). This equates to roughly one avoidable consultation for every 10 consultations if the consultation was with the regular GP. The results from the adjusted model translated to a mean reconsultation interval of 61 days when the patient saw their regular GP, and 56 days for any other GP.

The findings of the subgroup analyses in which patients were split into males and females (see Supplementary Tables S3 and S4), and locums or non-regular GPs were split by previous engagement with the practice (see Supplementary Tables S5 and S6), were consistent with those reported here. Locums with fewer previous appointments at the practice ordered significantly even fewer tests than locums with more previous engagement (see Supplementary Table S7).

## Discussion

### Summary

Regular GPs saw patients (receiving an antibiotic prescription) who were older and had more comorbidities than those seeing other GPs, which was expected as such patients are known to prioritise continuity. Adjustment for these factors was necessary to disentangle the influence of continuity and locum status. After adjustment, patients who saw a regular GP reconsulted after a significantly longer interval, hence reducing practice workload. This occurred compared with locums as well as with non-regular GPs, so the differentiating factor was continuity.

However, important differences in practice activities and clinical outcomes emerged between the three kinds of GP in this acute context. Locums ordered significantly fewer investigations, while non-regular GPs ordered more than regular GPs, possibly because locums do not expect to be able to follow-up the results of the tests, or potentially because of practice systems preventing locums from referring or ordering tests. Compared with seeing a regular GP, patients seeing locums or non-regular GPs were more likely to attend an A&E department without admission within 7 days. However, patients seeing locums or their regular GP were both less likely to have emergency admissions in the same week than those seeing non-regular GPs.

### Strengths and limitations

This study used consultations before the COVID-19 pandemic and used face-to-face GP consultations when these were standard, a limitation. Although there are now proportionately more remote consultations, large numbers of face-to-face GP consultations still take place and these findings will apply; however, the authors have no evidence for the effects of continuity in remote consultations or consultations with other professionals. The subsample analyses confirmed the results of the main study. Classifying GPs into three groups enabled separation of differences in outcomes that were owing to continuity or owing to locum status. Although patients were excluded if they had <4 consultations in 2 years, the mean annual face-to-face GP consultation rate for 2016–2017 was 3.3,^19^ meaning many patients with below average attendance rates would have been included.

The CPRD does not indicate who made the appointment or when, nor does it categorise severity of problems, so the use of a prescription of any antibiotic was used as a proxy for an acute consultation. There are known differences in prescribing between locums and regular GPs, with locums found to prescribe antibiotics 4% more,^14^ and there may still be case mix and severity differences between patients seeing locums and other GPs. Some of these prescriptions may be for chronic conditions. However, the 11% figure for the proportion of these ‘acute’ consultations in the current study that were with a locum was the same as a study that used a range of conditions to select acute consultations.^14^

The measure of continuity is binary, giving equal weight to a mixed group of GPs, from those who had only seen the patient twice in the past 2 years to GPs with higher levels of continuity. Some non-regular GPs may also have seen the patient several times, meaning the study may be underestimating the effects of continuity. It was not possible to tell whether same day A&E attendances occurred before or after the GP consultation, another limitation. It also was not possible to tell whether hospital outcomes were the result of GPs sending patients to A&E or for emergency admission.

### Comparison with existing literature

Previous research had found that, when compared with all permanent GPs, consultations with locums were associated with similar risks of hospital admissions, slightly higher A&E attendance, and fewer reconsultations within a week.^15^ The current study found that when locums are compared with GPs with continuity, there are greater differences in outcomes and, in this case, locums had a shorter reconsultation interval. Although the current study investigated acute consultations only, a larger study of 10 million GP consultations in the UK also found a longer reconsultation interval after patients saw a regular GP.^10^ They estimated that if all practices offered continuity at the level of the top decile performers and targeted these continuity consultations to their patients who were most vulnerable, practice workload could be reduced by up to 5.2%.

An association of reduced hospital admissions with better GP continuity is well established.^8^^,^^9^ For regular GPs, the results of this study fit with the finding that patients are more empowered/enabled to manage their own health,^20^ which may mean they do not need to attend A&E departments or reconsult. Despite perceptions that consultations with locums increase overall practice workload,^12^ this was only found when compared with practice GPs with continuity. All non-regular GPs had similarly shorter reconsultation intervals.

### Implications for research and practice

Using an acute subset of consultations might have been expected to reduce differences by type of GP. In the UK, there is a move towards patients consulting healthcare professionals without continuity for acute problems, based on the assumption that continuity is not needed for these. The current study shows that there were differences in important outcomes even for acute appointments.

With continuity, including from acute consultations, GPs gain better understanding of the patient and the context of their lives. This may allow GPs to evaluate risk with more precision and deal with more problems in a single consultation, hence avoiding unnecessary emergency hospital admissions and A&E use while also making more referrals. Non-regular GPs did not perform differently from locums on key outcomes including reconsultation interval and hospital admissions.

The current analysis, adjusted for various confounding factors, showed that if patient populations seen by different GP types were the same, patients without continuity would need more appointments. Despite recent changes, GPs remain the professional group with the most appointments in general practice, with thousands of consultations between GPs and patients without continuity, providing huge scope for improvement, thus reducing workload.

## Data

The data used in this study were accessed through the CPRD and are subject to protocol approval by ISAC, therefore they cannot be directly shared.

## References

[1] Adler R, Vasiliadis A, Bickell N (2010). The relationship between continuity and patient satisfaction: a systematic review. Fam Pract.

[2] Dossa AR, Moisan J, Guénette L (2017). Association between interpersonal continuity of care and medication adherence in type 2 diabetes: an observational cohort study. CMAJ Open.

[3] Christakis DA, Mell L, Wright JA (2000). The association between greater continuity of care and timely measles-mumps-rubella vaccination. Am J Public Health.

[4] Delgado J, Evans PH, Pereira Gray D (2022). Continuity of GP care for patients with dementia: impact on prescribing and the health of patients. Br J Gen Pract.

[5] Gaglioti AH, Li C, Baltrus PT (2023). Interpersonal primary care continuity for chronic conditions is associated with fewer hospitalizations and emergency department visits among Medicaid enrollees. J Am Board Fam Med.

[6] Pereira Gray D, Sidaway-Lee K, White E (2018). Continuity of care with doctors - a matter of life and death? A systematic review of continuity of care and mortality. BMJ Open.

[7] Baker R, Freeman GK, Haggerty JL (2020). Primary medical care continuity and patient mortality: a systematic review. Br J Gen Pract.

[8] Barker I, Steventon A, Deeny SR (2017). Association between continuity of care in general practice and hospital admissions for ambulatory care sensitive conditions: cross sectional study of routinely collected, person level data. BMJ.

[9] Tammes P, Purdy S, Salisbury C (2017). Continuity of primary care and emergency hospital admissions among older patients in England. Ann Fam Med.

[10] Kajaria-Montag H, Freeman M, Scholtes S (2024). Continuity of care increases physician productivity in primary care. Manage Sci.

[11] Tammes P, Morris RW, Murphy M, Salisbury C (2021). Is continuity of primary care declining in England? Practice-level longitudinal study from 2012 to 2017. Br J Gen Pract.

[12] Stringer G, Ferguson J, Walshe K (2023). Locum doctors in English general practices: evidence from a national survey. Br J Gen Pract.

[13] Grigoroglou C, Walshe K, Kontopantelis E (2022). Locum doctor use in English general practice: analysis of routinely collected workforce data 2017–2020. Br J Gen Pract.

[14] Borek AJ, Pouwels KB, van Hecke O (2022). Role of locum GPs in antibiotic prescribing and stewardship: a mixed-methods study. Br J Gen Pract.

[15] Grigoroglou C, Walshe K, Kontopantelis E (2024). Comparing the clinical practice and prescribing safety of locum and permanent doctors: observational study of primary care consultations in England. BMC Med.

[16] Herrett E, Gallagher AM, Bhaskaran K (2015). Data Resource Profile Data Resource Profile: Clinical Practice Research Datalink (CPRD). Int J Epidemiol.

[17] Zou G (2004). A modified Poisson regression approach to prospective studies with binary data. Am J Epidemiol.

[18] Williamson T, Eliasziw M, Fick GH (2013). Log-binomial models: exploring failed convergence. Emerg Themes Epidemiol.

[19] Kontopantelis E, Panagioti M, Farragher T (2021). Consultation patterns and frequent attenders in UK primary care from 2000 to 2019: a retrospective cohort analysis of consultation events across 845 general practices. BMJ Open.

[20] Howie JG, Heaney DJ, Maxwell M (1999). Quality at general practice consultations: cross sectional survey. BMJ.

